# Supplementary material for: Mesenchymal properties of iPSC-derived neural progenitors that generate undesired grafts after transplantation
Source: Commun Biol. 2023 Jun 7;6:611. doi: 10.1038/s42003-023-04995-9 (PMC10247757; doi:10.1038/s42003-023-04995-9)
Supplement: Supplementary file 2 — Supplementary Information [file 42003_2023_4995_MOESM2_ESM.pdf]

Supplementary Information

Supplementary Figure 1-13.

Supplementary Figure 1

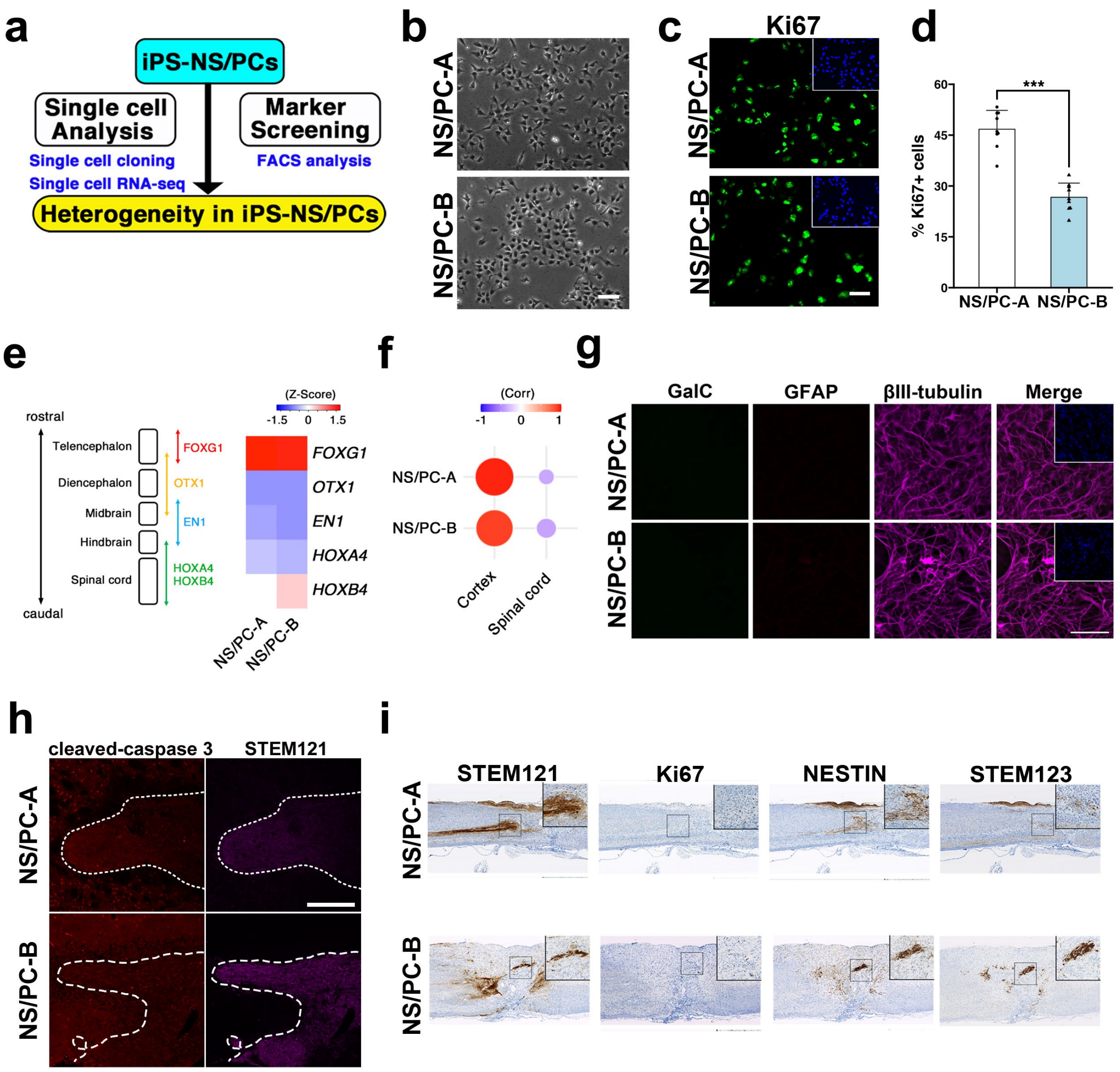

Supplementary Figure 1. NS/PCs lines derived from hiPSCs and histological features of the NS/PCs after transplantation into an injured spinal cord.

a) Overview of analyzing hiPSC-NS/PC heterogeneity. Single cell-based analyses and cell surface marker screening were applied to determine the cellular composition of parental hiPSC-NS/PCs.

b) Representative images of hiPSC-NS/PCs (NS/PC-A and NS/PC-B) used in this study. Scale bar, 100 μm.

c, d) Representative images (c) and quantification (d) of immunocytochemical analysis of hiPSC-NS/PCs (NS/PC-A and NS/PC-B) using antibody against Ki67. Scale bar, 50 μm.

e) Heatmap showing the gene expression of regional genes (FOXG1, OTX1, EN1, HOXA4, and HOXB4) in NS/PC-A and NS/PC-B.

f) Correlation plot comparing the transplanted cells (NS/PC-A and NS/PC-B) and publicly available dataset of human neuroepithelial stem cells isolated from the neocortex and spinal cord.

g) Differentiation capacity of hiPSC-NS/PCs as assessed by the expression of neuronal (βIII-tubulin) and glial (GFAP, GalC) markers after 14 days of differentiation. Scale bar, 100 μm.

h) Representative images of cleaved-caspase 3 expression in $STEM121^{+}$ grafts at 3 months after transplantation into the striatum. There is no apparent difference in apoptotic cell population between NS/PC-A and NS/PC-B. Scale bar, 200μm.

i) Representative tissue sections of the injured spinal cord at 3 months after transplantation of each hiPSC-NS/PC line. Grafted cells were evaluated by the expression of STEM121, Ki67, NESTIN, and human-specific GFAP (STEM123). Insets: higher magnification images of the boxed regions. Scale bar, 500 μm.

Supplementary Figure 2

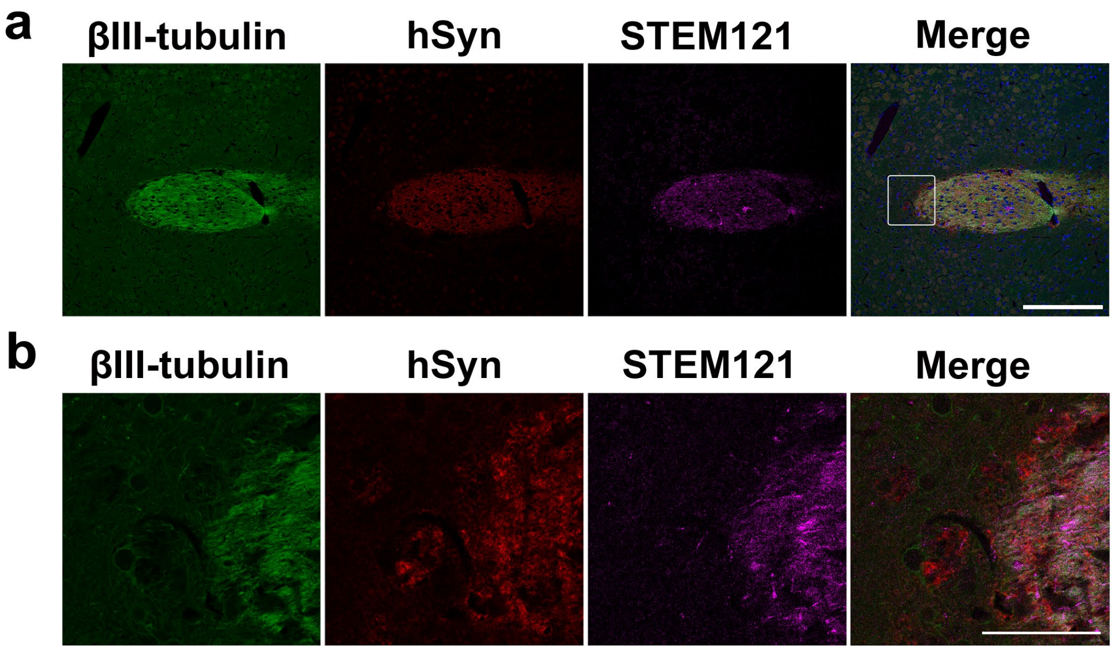

Supplementary Figure 2. Histological evaluation of hiPSC-NS/PC-derived grafts. a, b) Representative images of human synaptophysin expression in STEM121^{+} grafts derived from NS/PC-A at 3 months after transplantation into the striatum. Neuronal differentiation capacity of NS/PCs-A in the graft was evaluated using antibodies against βIII-tubulin, human-specific Synapsin (hSyn), and STEM121. The panels in (b) were higher magnification of box region in (a). Scale bars, (a): 200 μm, (b): 50μm.

Supplementary Figure 3

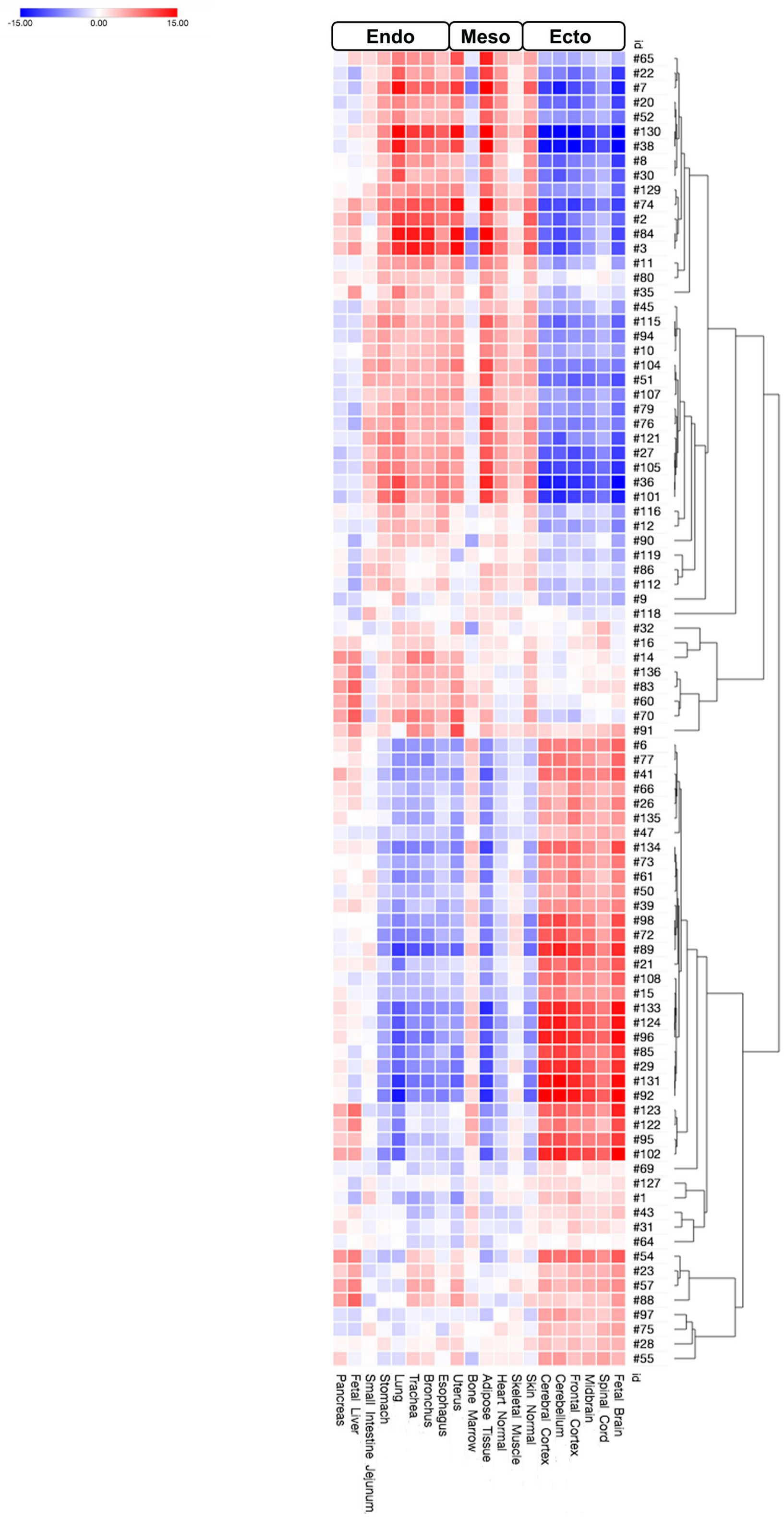

Supplementary Figure 3. Correlation of the gene expression profiles of single cell-derived NS/PCs and somatic organs.

Correlation of gene expression profiles obtained by microarray analysis of scNS/PCs with public datasets of somatic tissues. The color shows the z-value for correlation significance.

Supplementary Figure 4

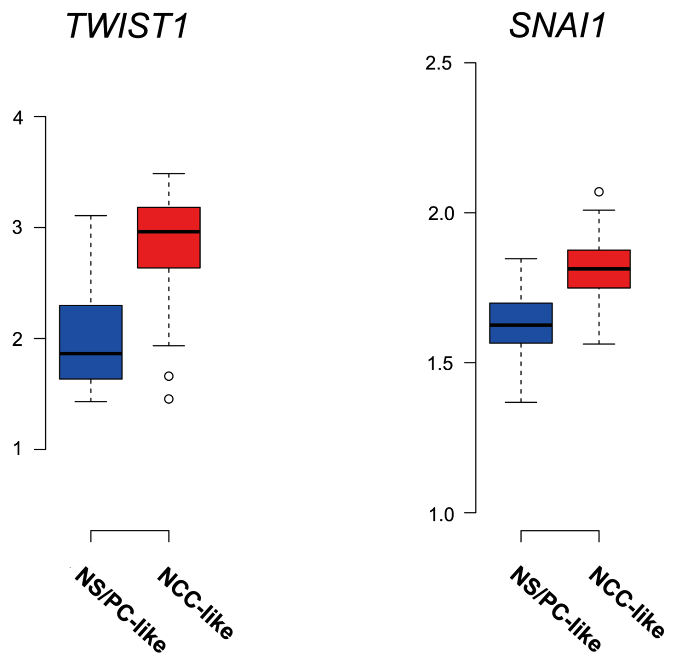

Supplementary Figure 4. Expression of genes associated with tumorigenicity of hiPSC-NS/PCs in scNS/PCs.

Boxplots of *TWIST1* and *SNAI1* expression in NS/PC- and NCC-like scNS/PCs. Centerlines show medians, box limits indicate the 25th and 75th percentiles as determined by R software, whiskers extend 1.5 times the interquartile range from the 25th and 75th percentiles, and outliers are represented by dots.

Supplementary Figure 5

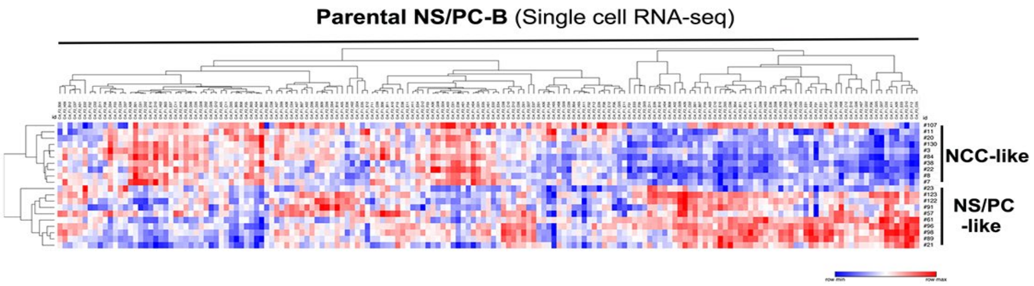

Supplementary Figure 5. Gene expression profiles of scNS/PCs.

Correlation of gene expression profiles obtained by microarray analysis of representative NS/PC- and NCC-like scNS/PCs with expression data obtained by single cell RNA-seq of parental NS/PCs. The color shows the z-value for correlation significance.

Supplementary Figure 6

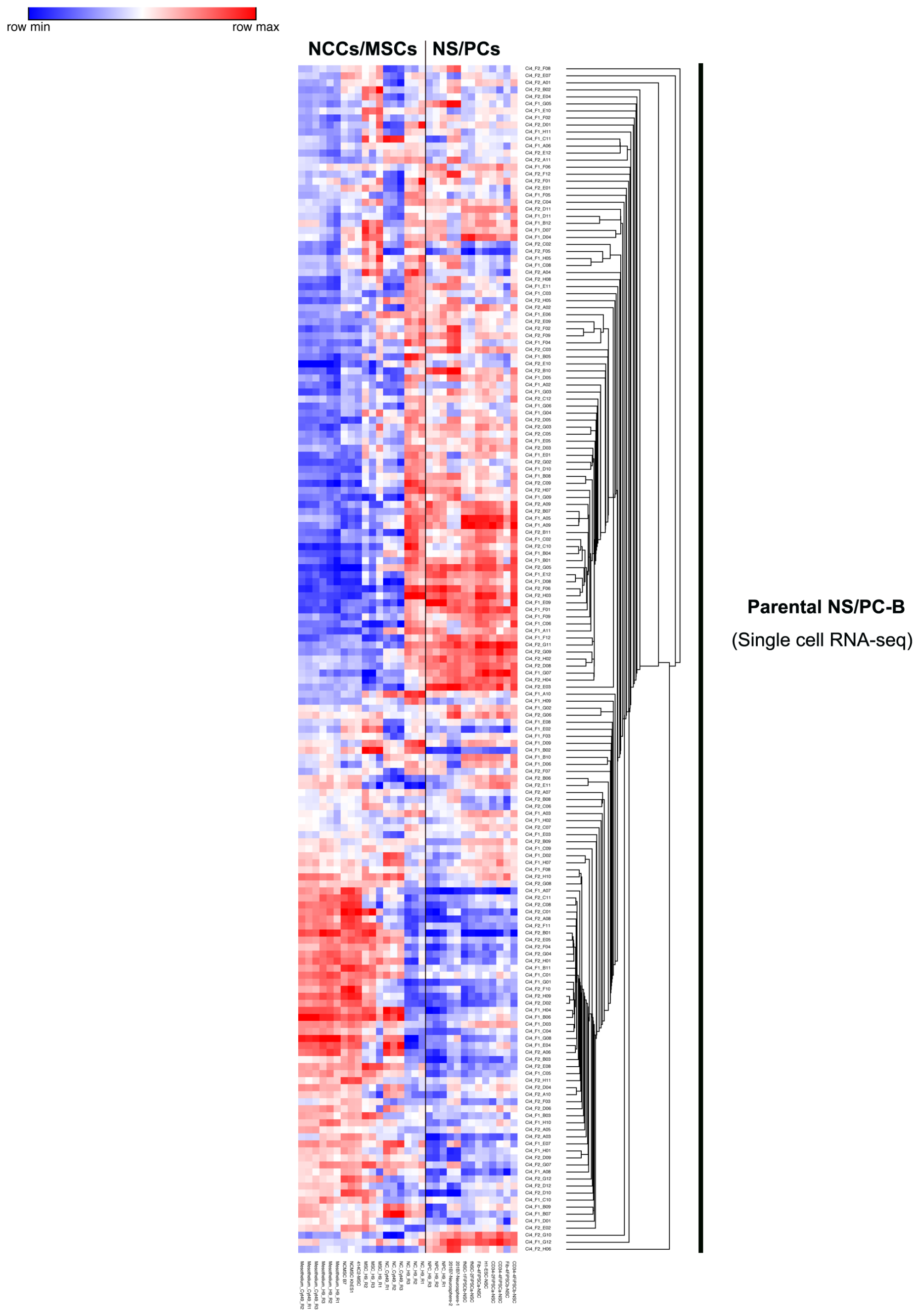

Supplementary Figure 6. Correlation of the gene expression profile from single cell RNA-seq data of NS/PCs with public datasets of NS/PCs, NCCs, and MSC.
Correlation of gene expression profiles obtained by single cell RNA-seq of parental NS/PCs with gene expression of NS/PCs, NCCs, and MSCs available from public datasets together with clustering. The color shows the z-value for correlation significance.

Supplementary Figure 7

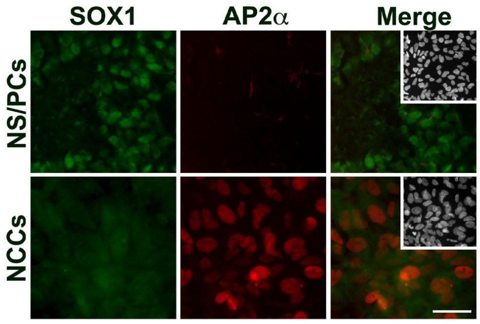

Supplementary Figure 7. Expression of SOX1 and AP2α expression on NS/PCs and iPSC-NCCs.

Representative images of SOX1 and AP2α expression on NS/PCs and iPSC-NCCs. Inset: Hoechst nuclear staining of the same field. Scale bar, 50 μm.

Supplementary Figure 8

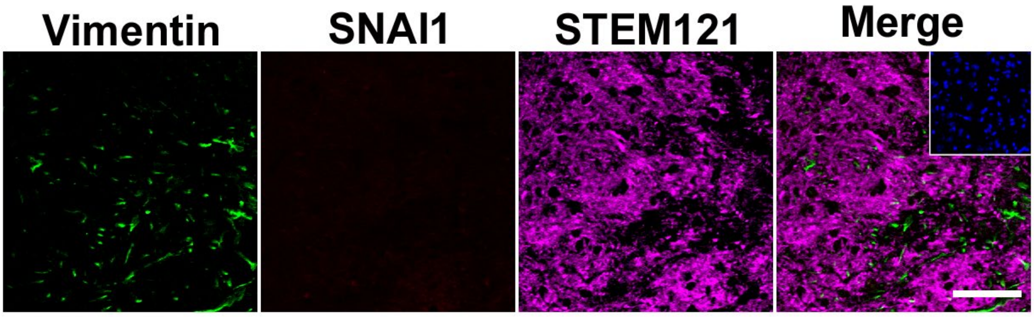

Supplementary Figure 8. Expression of Vimentin and SNAI1 in the NS/PC-B-derived grafts in the striatum.

Representative images of Vimentin and SNAI1 expression in STEM121^{+} grafts at 3 months after transplantation. Inset: Hoechst nuclear staining of the same field. Scale bar, 50 μm.

Supplementary Figure 9

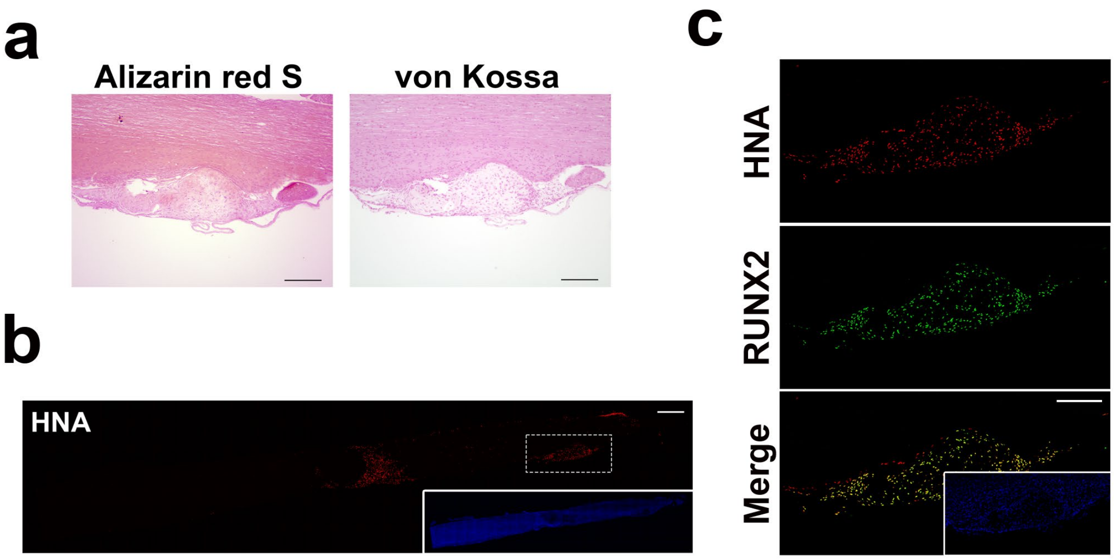

Supplementary Figure 9. Expression of RUNX2 in the NS/PC-B-derived grafts in the injured spinal cord.

a) Evaluation of calcium deposition in the grafts by Alizarin red S and von Kossa staining. Calcium deposition was not detected in the graft. Scale bars, 200 µm.

b) Representative images of NS/PC-B derived grafts visualized by HNA-expression (red) at 6 months after transplantation into an injured spinal cord. The area enclosed by the dotted line is region indicated by the analysis in (b). Inset: Hoechst nuclear staining of the same field. Scale bar, 500 µm.

c) Representative images of RUNX2 expression (green) in the NS/PC-B-derived grafts. Inset: Hoechst nuclear staining of the same field. Scale bar, 200 µm.

Supplementary Figure 10

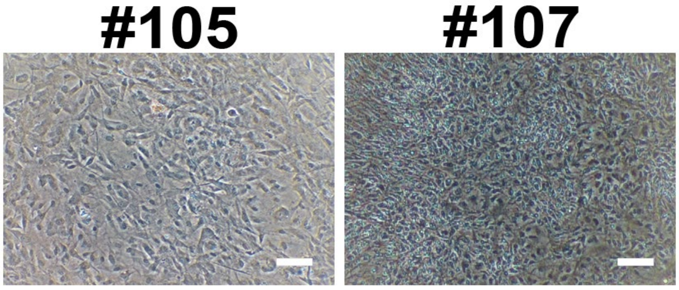

Supplementary Figure 10. Adipogenic differentiation capacity of NCC-like scNS/PCs.

Oil Red O staining of NCC-like (#105 and #107) scNS/PCs after adipogenic differentiation. No lipid droplet was observed in the scNS/PCs after the adipogenic differentiation. Scale bar, 100 μm.

Supplementary Figure 11

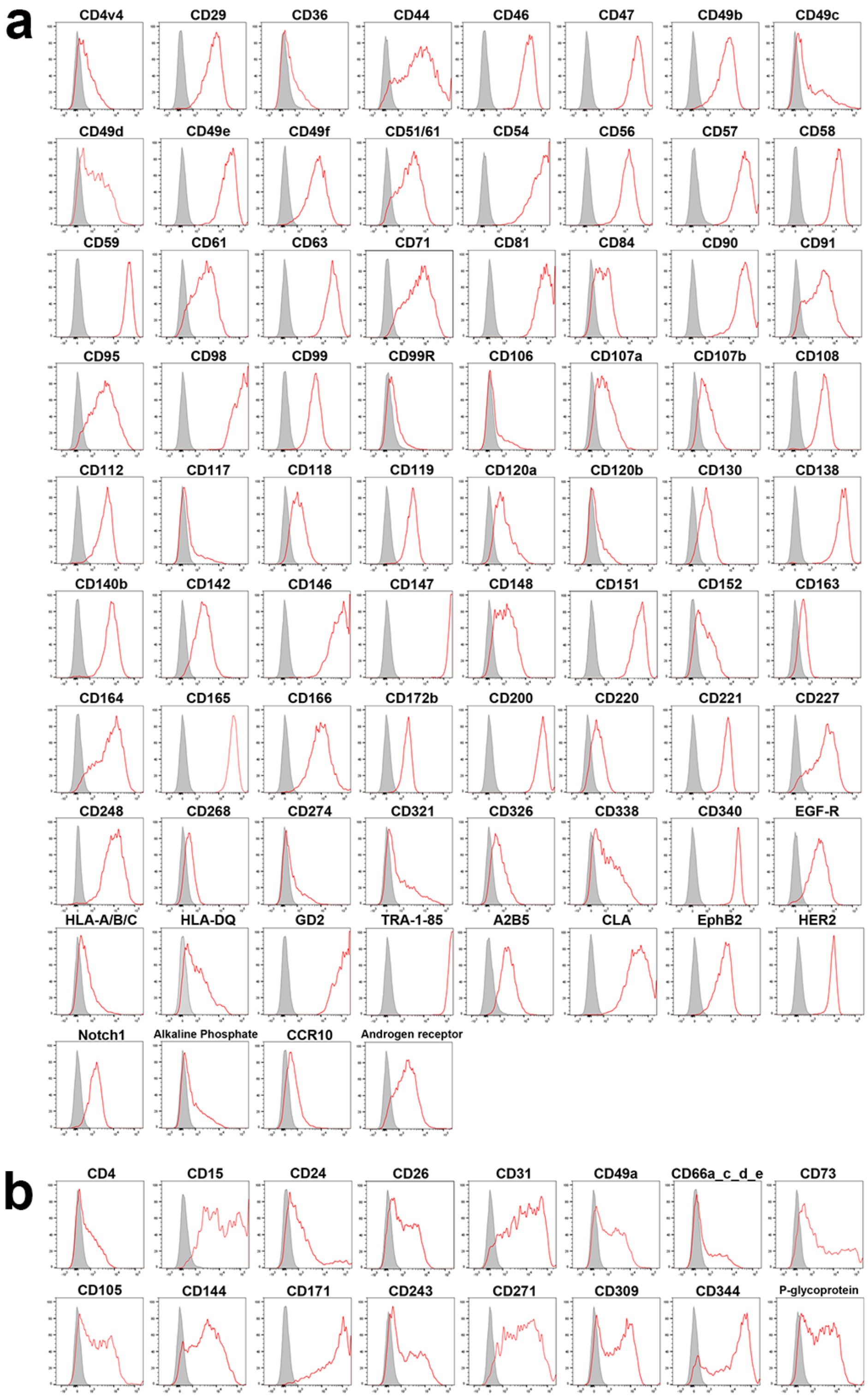

Supplementary Figure 11. Examination of surface markers on NS/PC-B by BD Lyoplate.

Fluorescence-activated cell sorting (FACS) analysis of cell surface markers on NS/PC-B cells. FACS analysis of positive markers (a) and positive markers with subpopulations (b) on NS/PC-B cells are shown.

Supplementary Figure 12

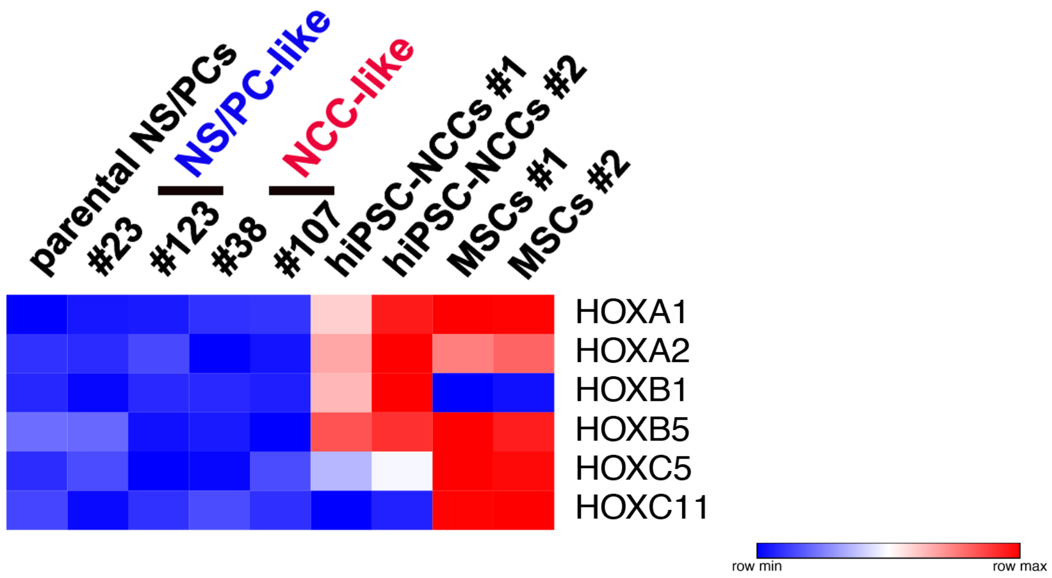

Supplementary Figure 12. Heatmap displaying expression of HOX genes in parental NS/PC, scNS/PCs, hiPSC-NCCs, and MSCs.

Supplementary Figure 13

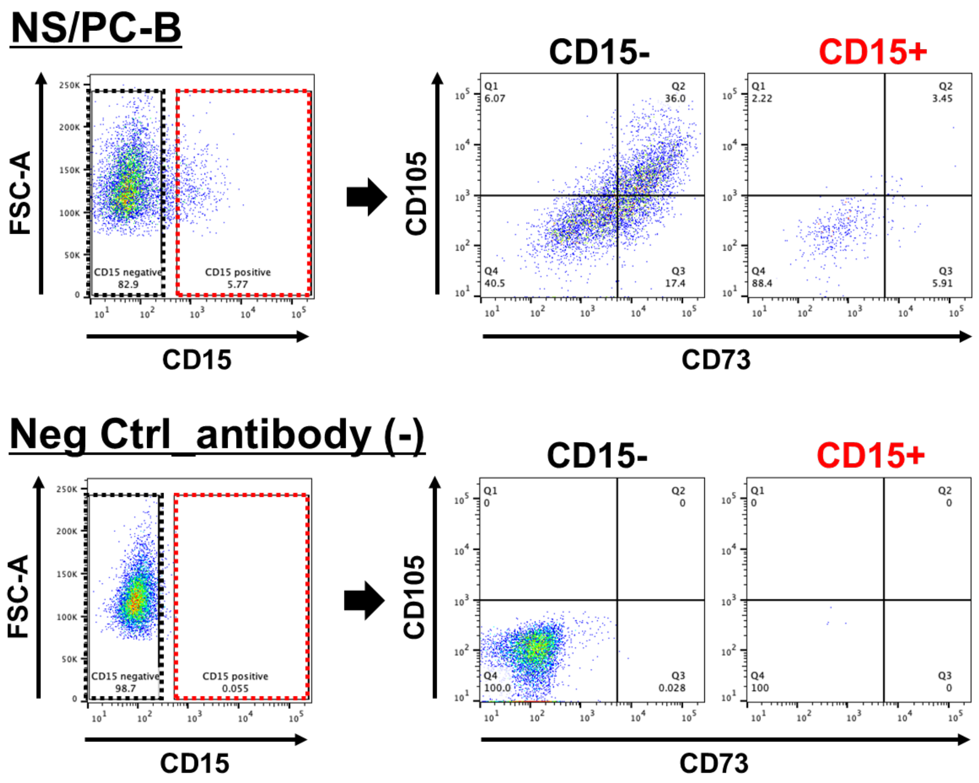

Supplementary Figure 13. Gating strategy.

The gating strategy for FACS sorting (Fig. 6) is shown.
