# Supplementary material for: Mesenchymal properties of iPSC-derived neural progenitors that generate undesired grafts after transplantation
Source: Commun Biol. 2023 Jun 7;6:611. doi: 10.1038/s42003-023-04995-9 (PMC10247757; doi:10.1038/s42003-023-04995-9)
Supplement: Supplementary file 5 — Reporting Summary [file 42003_2023_4995_MOESM5_ESM.pdf]

nature portfolio

Corresponding author(s): JUN KOHYAMA

Last updated by author(s): May 24, 2023

Reporting Summary

Nature Portfolio wishes to improve the reproducibility of the work that we publish. This form provides structure for consistency and transparency in reporting. For further information on Nature Portfolio policies, see our Editorial Policies and the Editorial Policy Checklist.

Statistics

For all statistical analyses, confirm that the following items are present in the figure legend, table legend, main text, or Methods section.

n/a	Confirmed	
☐	☒	The exact sample size (*n*) for each experimental group/condition, given as a discrete number and unit of measurement
☐	☒	A statement on whether measurements were taken from distinct samples or whether the same sample was measured repeatedly
☐	☒	The statistical test(s) used AND whether they are one- or two-sided<br>*Only common tests should be described solely by name; describe more complex techniques in the Methods section.*
☒	☐	A description of all covariates tested
☒	☐	A description of any assumptions or corrections, such as tests of normality and adjustment for multiple comparisons
☐	☒	A full description of the statistical parameters including central tendency (e.g. means) or other basic estimates (e.g. regression coefficient) AND variation (e.g. standard deviation) or associated estimates of uncertainty (e.g. confidence intervals)
☐	☒	For null hypothesis testing, the test statistic (e.g. *F*, *t*, *r*) with confidence intervals, effect sizes, degrees of freedom and *P* value noted<br>*Give P values as exact values whenever suitable.*
☒	☐	For Bayesian analysis, information on the choice of priors and Markov chain Monte Carlo settings
☒	☐	For hierarchical and complex designs, identification of the appropriate level for tests and full reporting of outcomes
☒	☐	Estimates of effect sizes (e.g. Cohen's *d*, Pearson's *r*), indicating how they were calculated

Our web collection on statistics for biologists contains articles on many of the points above.

Software and code

Policy information about availability of computer code

Data collection	Microarray analysis: RNA quality was assessed using an Agilent 2100 Bioanalyzer (Agilent Technologies). Total RNA (200 ng) was reverse transcribed, labeled with biotin using a Target Amp-Nano Labeling Kit for Illumina Expression BeadChip (Epicentre, Illumina), and hybridized to a HumanHT-12_v4_BeadChip (Illumina) in accordance with the manufacturer's instructions. The array was washed and stained using an Illumina gene expression kit. Raw intensity values were acquired using an iScan microarray scanner (Illumina). Raw probe intensity files were exported using Illumina GenomeStudio gene expression software (v1.9.0).<br>RNA-Seq:Samples for RNA-seq were prepared using a TruSeq RNA Sample Prep Kit (Illumina) in accordance with the manufacturer's protocol. The sequencing library was sequenced on a HiSeq 2500 (Illumina). Base calling and chastity filtering were performed using Real-Time Analysis Software version 1.18.61. Raw reads were mapped to the reference genome hg19 using sailfish (v0.7.6) with default settings.<br>Single cell RNA-Seq: The DNA sequencing library was analyzed with the massively parallel sequencer HiSeq 2500. Raw reads were trimmed by read quality and read length using Trimmomatic software (v0.33). Trimmed reads were aligned to the reference genome hg19 using sailfish (v0.7.6) with default settings. Samples were filtered by the following parameters and used for analysis: read number > 1 million, aligned rate > 70%, and detected gene number > 5000.
Data analysis	R software: v4.1.1<br>Microarray analysis:Genes differentially expressed by 1.2-fold between NS/PC-like scNS/PCs and NCC-like scNS/PCs were extracted and applied to GO analysis using DAVID Bioinformatics Resources (http://david.ncifcrf.gov). Box plot evaluation of gene expression was performed using BoxPlotR (http://shiny.chemgrid.org)<br>Correlation analysis:The correlation of differentially expressed genes in individual cells with publicity available datasets for representative

tissues or cells was evaluated using ExAtlas (https://lgsun.irp.nia.nih.gov/exatlas/)
FACS:Data were analyzed using FlowJo, version 7.6.

For manuscripts utilizing custom algorithms or software that are central to the research but not yet described in published literature, software must be made available to editors and reviewers. We strongly encourage code deposition in a community repository (e.g. GitHub). See the Nature Portfolio guidelines for submitting code & software for further information.

Data

Policy information about availability of data

All manuscripts must include a data availability statement. This statement should provide the following information, where applicable:

- Accession codes, unique identifiers, or web links for publicly available datasets
- A description of any restrictions on data availability
- For clinical datasets or third party data, please ensure that the statement adheres to our policy

GSE166134

Human research participants

Policy information about studies involving human research participants and Sex and Gender in Research.

Reporting on sex and gender	*Use the terms sex (biological attribute) and gender (shaped by social and cultural circumstances) carefully in order to avoid confusing both terms. Indicate if findings apply to only one sex or gender; describe whether sex and gender were considered in study design whether sex and/or gender was determined based on self-reporting or assigned and methods used. Provide in the source data disaggregated sex and gender data where this information has been collected, and consent has been obtained for sharing of individual-level data; provide overall numbers in this Reporting Summary. Please state if this information has not been collected. Report sex- and gender-based analyses where performed, justify reasons for lack of sex- and gender-based analysis.*
Population characteristics	*Describe the covariate-relevant population characteristics of the human research participants (e.g. age, genotypic information, past and current diagnosis and treatment categories). If you filled out the behavioural & social sciences study design questions and have nothing to add here, write "See above."*
Recruitment	*Describe how participants were recruited. Outline any potential self-selection bias or other biases that may be present and how these are likely to impact results.*
Ethics oversight	*Identify the organization(s) that approved the study protocol.*

Note that full information on the approval of the study protocol must also be provided in the manuscript.

Field-specific reporting

Please select the one below that is the best fit for your research. If you are not sure, read the appropriate sections before making your selection.

☒ Life sciences ☐ Behavioural & social sciences ☐ Ecological, evolutionary & environmental sciences

For a reference copy of the document with all sections, see nature.com/documents/nr-reporting-summary-flat.pdf

Life sciences study design

All studies must disclose on these points even when the disclosure is negative.

Sample size	We obtained 90 single-cell derived NS/PC clones. No statistical method was utilized to predetermine the sample size. Sample size was determined by the number of available clones.
Data exclusions	No data were excluded from the analyses.
Replication	All replicates were biological replicates. The number of replicates were indicated in the manuscript.
Randomization	All samples were randomly allocated into experimental groups.
Blinding	Investigators were blinded during data collection or data analysis.

Reporting for specific materials, systems and methods

We require information from authors about some types of materials, experimental systems and methods used in many studies. Here, indicate whether each material, system or method listed is relevant to your study. If you are not sure if a list item applies to your research, read the appropriate section before selecting a response.

Materials & experimental systems

n/a	Involved in the study
☐	☒ Antibodies
☐	☒ Eukaryotic cell lines
☒	☐ Palaeontology and archaeology
☐	☒ Animals and other organisms
☒	☐ Clinical data
☒	☐ Dual use research of concern

Methods

n/a	Involved in the study
☒	☐ ChIP-seq
☐	☒ Flow cytometry
☒	☐ MRI-based neuroimaging

Antibodies

Antibodies used

All antibodies used in this study are detailed in "Material and Methods" section. For immunochemistry, anti-SOX1 (R&D Systems, AF3369; 1:500), anti-SOX2 (R&D Systems, MAB2018; 1:500), anti-human Nestin (Immuno-Biological Laboratories Co., 18741; 1:500 and Millipore, MAB5326; 1:200), anti-SOX9 (Santa Cruz Biotechnology, sc-20095; 1:200), anti-βIII-tubulin (Sigma-Aldrich, T8660; 1:500), anti-AP2α (Santa Cruz Biotechnology, sc-12726; 1:50), anti- nELAVL (Thermo Fisher Scientific, A21271; 1:50), anti-human nuclear antigen (HNA; Millipore, MAB4383; 1:100), anti-human cytoplasm (STEM121, Takara Bio, Y40410; 1:100), anti-human Vimentin NL493-conjugated rat IgG2a, and anti-human Snail NL557-conjugated goat IgG (Human EMT 3-Color Immunocytochemistry Kit, R&D Systems, SC026) were used. For flow cytmetic analysis, the BD Lyoplate™ Human Cell Surface Marker Screening Panel BD Biosciences; 560747), PSA-NCAM (Millipore, MAB5324), PSA-NCAM-APC (Miltenyi Biotec, 130-093-273), CD133-APC (Miltenyi Biotec, 130-090-826), CD15-Brilliant Violet 421 (BioLegend, 323039), CD49α-FITC (BioLegend, 328308), CD73-PE-Cy7, (BioLegend, 127224), and CD105-APC (BioLegend, 323208) were used.

Validation

All antibodies are commercially available and were validated by the manufactures.

Eukaryotic cell lines

Policy information about cell lines and Sex and Gender in Research

Cell line source(s)

Human iPSC lines 1210B2 and 1231A3 were established from PBMCs using episomal vectors at the Center for iPS Cell Research and Application (CiRA). Human iPSC line 201B7 was established from dermal fibroblasts using retrovirus vectors at Kyoto university. Human iPSC line WD39 was established from dermal fibroblasts using retrovirus vectors at Keio university. Human iPSC-derived NS/PC line AF22 and human ESC-derived NS/PC line AF23 were established at University of Cambridge.

Authentication

Dr. Yamanaka at CiRA supplied the iPSC lines. Dr. Austin Smith at Cambridge University supplied the NS/PC lines AF22 and AF23.

Mycoplasma contamination

All cell lines were negative for mycoplasma contamination.

Commonly misidentified lines
(See ICLAC register)

N/A

Animals and other research organisms

Policy information about studies involving animals; ARRIVE guidelines recommended for reporting animal research, and Sex and Gender in Research

Laboratory animals

9-week-old NOD/Shi-SCID, IL-2Rγ-null (NOG) mice and NOD/SCID mice were used in this study.

Wild animals

No wild animals were utilized in this study.

Reporting on sex

Female mice were used in this study. (Sex was not considered in study design. Our laboratory has been using female mice for study.)

Field-collected samples

The study did not involve samples collected from the field.

Ethics oversight

All animal experiments were performed in accordance with the Guide for Care and Use of Laboratory Animals of the Central Institute for Experimental Animals (CIEA; Kanagawa, Japan). The experimental protocols were approved by the CIEA Animal Care Committee (Permit number: 11029A) and Keio University School of Medicine (Tokyo, Japan) (Permit number: 16-096-25).

Note that full information on the approval of the study protocol must also be provided in the manuscript.

Flow Cytometry

Plots

Confirm that:

☐ The axis labels state the marker and fluorochrome used (e.g. CD4-FITC).

☒ The axis scales are clearly visible. Include numbers along axes only for bottom left plot of group (a 'group' is an analysis of identical markers).

☒ All plots are contour plots with outliers or pseudocolor plots.

☒ A numerical value for number of cells or percentage (with statistics) is provided.

Methodology

Sample preparation	Cells were suspended in PBS containing 0.5% bovine serum albumin and 2 mM EDTA (pH 8.0) and stained for 30 minutes on ice in the dark with fluorescent dye-conjugated antibodies.
Instrument	FACSVerse flow cytometer (BD Biosciences), LSRFortessa flow cytometer (BD Biosciences), FACSAria cell sorter (BD Biosciences)
Software	FlowJo, version 7.6 (TreeStar)
Cell population abundance	*Describe the abundance of the relevant cell populations within post-sort fractions, providing details on the purity of the samples and how it was determined.*
Gating strategy	Debris, doublets were excluded by forward scatter/side scatter gating. 7-AAD fluorescence was measured and a live gate was difined that excluded cells positive for 7-AAD. Additional gates were defined as positive for each marker according to the isotype control fluorescence intensity.

☒ Tick this box to confirm that a figure exemplifying the gating strategy is provided in the Supplementary Information.
